# Email Between Patient and Provider: Assessing the Attitudes and Perspectives of 624 Primary Health Care Patients

**DOI:** 10.2196/medinform.5853

**Published:** 2016-12-22

**Authors:** Puneet Seth, Mohamed Ismail Abu-Abed, Vikram Kapoor, Kathryn Nicholson, Gina Agarwal

**Affiliations:** ^1^ Department of Family Medicine Schulich School of Medicine & Dentistry Western University London, ON Canada; ^2^ Division of Hospital Medicine Woodstock General Hospital Woodstock, ON Canada; ^3^ Brampton Civic Hospital Brampton, ON Canada; ^4^ Department of Epidemiology & Biostatistics Schulich School of Medicine & Dentistry Western University London, ON Canada; ^5^ Family Medicine Residency Program Department of Family Medicine McMaster University Hamilton, ON Canada

**Keywords:** electronic mail, email, communication, primary health care, surveys, patient engagement

## Abstract

**Background:**

Email between patients and their health care providers can serve as a continuous and collaborative forum to improve access to care, enhance convenience of communication, reduce administrative costs and missed appointments, and improve satisfaction with the patient-provider relationship.

**Objective:**

The main objective of this study was to investigate the attitudes of patients aged 16 years and older toward receiving email communication for health-related purposes from an academic inner-city family health team in Southern Ontario. In addition to exploring the proportion of patients with a functioning email address and interest in email communication with their health care provider, we also examined patient-level predictors of interest in email communication.

**Methods:**

A cross-sectional study was conducted using a self-administered, 1-page survey of attitudes toward electronic communication for health purposes. Participants were recruited from attending patients at the McMaster Family Practice in Hamilton, Ontario, Canada. These patients were aged 16 years and older and were approached consecutively to complete the self-administered survey (N=624). Descriptive analyses were conducted using the Pearson chi-square test to examine correlations between variables. A logistic regression analysis was conducted to determine statistically significant predictors of interest in email communication (yes or no).

**Results:**

The majority of respondents (73.2%, 457/624) reported that they would be willing to have their health care provider (from the McMaster Family Practice) contact them via email to communicate health-related information. Those respondents who checked their personal email more frequently were less likely to want to engage in this electronic communication. Among respondents who check their email less frequently (fewer than every 3 days), 46% (37/81) preferred to communicate with the McMaster Family Practice via email.

**Conclusions:**

Online applications, including email, are emerging as a viable avenue for patient communication. With increasing utility of mobile devices in the general population, the proportion of patients interested in email communication with their health care providers may continue to increase. When following best practices and appropriate guidelines, health care providers can use this resource to enhance patient-provider communication in their clinical work, ultimately leading to improved health outcomes and satisfaction with care among their patients.

## Introduction

The use of the Internet and electronic communication for day-to-day purposes is becoming an increasingly ubiquitous resource in many developed countries around the world [1]. The use of technology and electronics in health care delivery is also continuing to rise in prevalence [2-7]. Among other modalities [8], email between patients and their health care providers can serve as a continuous and collaborative forum to improve access to care, enhance convenience of communication outside of traditional office hours, reduce administrative costs and missed appointments, and improve satisfaction with the patient-provider relationship [2,9-14]. A systematic review conducted by Ye et al (2010) included content analyses of email messages between patients and health care providers and indicated that emails were commonly used for medical information exchange, medical condition or update, medication information, and subspecialty evaluation [12].

The benefits and risks associated with using email communication have been well-articulated in previous literature [2,6,7,9,14,15]. The potential advantages of email in delivering health care include (1) increased convenience for patients and providers (eg, time savings, avoiding need for in-person visit) [2,9-11]; (2) the continuous recording of health-related information (eg, tests results, addresses and telephone numbers of referrals, postoperative instructions) [2,10]; (3) increased opportunity for information sharing (eg, sending educational material relevant to their health) [2,10]; and (4) a user-friendly medium for patients to ask clarification questions after a face-to-face consultation [2,12]. However, there is concern from health care providers that improper use of this resource may hinder the patient-provider relationship [2,4,5], become a source of legal liability [12,15], increase the risk of diagnostic or communication errors [2,15], highlight social disparities among patients [2,14,16], and threaten patient privacy [2,4,12,15,17-19]. Providers have also been wary of adopting email as a major mode of communication with their patients, citing concerns of reimbursement, inundation with email, time demands, and the possibility of dealing with trivial issues or topics that are inappropriate to manage over email [4,17,19-21]. Despite these concerns, some studies have indicated that the email medium has promise in improving communication and access in health care. For example, patients tended to use the format appropriately by avoiding emergent issues, limiting the content to medical and administrative-oriented topics (eg, arranging appointments), and including only one request per email [9,12,22,23].

The main objective of this study, conducted as part of a Quality Assurance project at McMaster Family Practice, is to investigate the attitudes of patients aged 16 years and older toward receiving email communication for health-related purposes from an academic inner-city family health team in Southern Ontario. This was achieved through the development and distribution of a questionnaire by the study authors that identified patient concerns around email communication, their willingness to use this modality for communication from the clinic, and what specific purposes they felt would be most useful.

## Methods

### Setting and Study Sample

The project took place at McMaster Family Practice in Hamilton, Ontario, Canada. McMaster Family Practice is a large academic family medicine clinic situated in the downtown of an urban region that provides a full range of comprehensive primary care, with a particular focus on inner city health issues. Patients aged 16 years and older, who attended the clinic, were eligible to participate in the survey. Patient recruitment occurred during the time of checking in for a clinic visit with the medical office assistant. Patients meeting eligibility criteria (greater than 16 years of age, fluent in English, and without any diagnosis of cognitive impairment) were offered the opportunity to participate in the study. If they agreed, they were provided with a clipboard with the questionnaire and a pen—there was no digital modality offered for this questionnaire. Patients who agreed to complete the pseudonymous survey were compensated for their participation with a small treat (value less than Can $1), and completed the survey in the practice waiting room before their health care encounter. Approval for the project was granted by the Hamilton Integrated Research Ethic Board.

### Study Design and Data Collection

The study was a cross-sectional, self-administered survey of patients who met the inclusion criteria at the date of data collection. The survey instrument was a 1-page, 2-sided document that was developed by the authors following a literature review and discussion (see Multimedia Appendix 1). In addition to demographic characteristics, respondents were asked about their satisfaction or dissatisfaction of the potential to use email communication with their health care provider. Responses from completed surveys were entered into an electronic database for analysis. Surveys were completed anonymously, with only their personal identifiers (the first three digits of the patient’s residential postal code) and patient age at date of data collection.

### Data Analysis

Descriptive analyses were conducted to examine participant characteristics, frequencies of responses, and relationships between key variables. A Pearson chi-square test was conducted to explore correlations between variables, and a stepwise logistic regression analysis was conducted to identify the independent variables that were statistically significant predictors of the dependent variable, which was patient interest in email communication (yes or no). The distribution of independent and dependent variables was explored before analysis. The significance level was set to .05, while case-wise deletion was used for missing data. All analyses were conducted using SPSS Version 19.

## Results

### Participant Characteristics

A summary of all participant characteristics and demographics is presented in Table 1. Overall, 49.7% (310/624) of respondents were female and 17.6% (110/624) were between the ages of 35 and 44 years. Slightly less than half of the respondents had completed university-level education (43.1%, 269/624) and were employed at the time of the study (47.6%, 297/624). While 87.5% (546/624) of respondents stated that they had a personal email address, 73.2% (457/624) of patients stated that they would be willing to have health-related email communication with the McMaster Family Practice.

**Table 1:** Characteristics and demographics of study participants (N=624).

Patient Characteristics		n (%)
**Sex**		
	Male	186 (29.8)
	Female	310 (49.7)
	Not specified	128 (20.5)
**Age, years**		
	16-24	47 (7.5)
	25-34	102 (16.3)
	35-44	110 (17.6)
	45-54	105 (16.8)
	55-64	86 (13.8)
	65-74	51 (8.2)
	75-84	26 (4.2)
	85-94	3 (0.5)
	Not specified	94 (15.1)
**Education level**		
	Less than high school	16 (2.6)
	High school	75 (12.0)
	College	129 (20.7)
	Undergraduate	134 (21.5)
	Postgraduate	135 (21.6)
	Not specified	135 (21.6)
**Employment status**		
	Employed	297 (47.6)
	Retired	98 (15.7)
	Unemployed	67 (10.7)
	Not specified	162 (26.0)

### Willingness to Use Email Communication

The correlation between how often a participant checked their email and their willingness to receive email communication was assessed and is presented in Table 2. A total of 90.6% (414/457) of respondents who checked their email frequently (at least once every 3 days) were willing to be contacted by email, as compared to 45.7% (37/81) of participants who checked their email less frequently (*P*<.001). Interestingly, the willingness to be contacted did not vary by patient age (*P*=.30) or patient sex (*P*=.95). In examining the influence of education level, 88.1% (119/135) of patients who did have a postgraduate education were open to email communication, while 77.0% (271/352) of respondents who did not have a postgraduate education were still open to email communication (*P*<.001). A total of 70.0% (437/624) of patient respondents did not have an interest in SMS text messaging (short message service, SMS) communication. This trend was evident regardless of age group. When asked about privacy concerns, 63.3% (395/624) of respondents were not concerned or only somewhat concerned. However, 24.7% (154/624) of patients stated that privacy was a serious concern and the remaining 12.0% (75/624) of respondents were unsure or undecided. Among patients that were not concerned or only somewhat concerned about privacy, 87.7% (270/308) were willing to be contacted by email, as compared to 74.2% (89/120) of respondents who were concerned or very concerned about privacy (*P*<.001).

In the logistic regression analysis, which determined predictors of respondent interest in health-related email communication, 3 independent variables were found to be statistically significant (*P*<.05). The final model is presented in Table 3. Among those patients who accepted text messages, there was a 3.7-fold increase in odds of whether these patients would also want to utilize email communication (*P*=.002), holding all other variables constant. Among patients who utilized personal email, there was an 8.3-fold increase in odds of whether the patient would also want to utilize email communication with their health care provider (*P*=.03), holding all other variables constant. Finally, patients who checked their email frequently were 58% less likely to be interested in email communication (*P*<.001), holding all other variables constant.

**Table 2:** Variable correlation with participant interest in health-related email communication.

Independent variable	Chi square test *P* value (degrees of freedom)
Concerned about privacy	<.001^a^ (5)
Concerned about junk mail	.45 (4)
Email benefit	<.001^a^ (4)
Frequently checks email	<.001^a^ (4)
Forgetting appointments	.01^b^ (5)
Overall satisfaction with current communication	.10 (4)

^a^Statistically significant, *P*<.01.

^b^Statistically significant, *P*<.05.

**Table 3:** Logistic regression analysis examining predictor variables of participant interest in health-related email communication.

Independent Variable	exp (B)^a^	*P* value^b^
Age category	1.16	.16
Sex	1.15	.67
More education	0.00	>.99
Less education	0.00	>.99
Employed	0.73	.33
Use of text messaging	3.72	<.001^c^
Frequently checks email	0.42	<.001^c^
Personal email	8.29	.03^d^
Overall satisfaction with current communication	1.00	>.99

^a^Exponentiation of the B coefficient (odds ratio).

^b^Controlling for all other independent variables in the model.

^c^Statistically significant, *P*<.01.

^d^Statistically significant, *P*<.05.

## Discussion

### Principal Findings

The vast majority of respondents (73.2%,457/624) reported willingness to communicate electronically with their family practice for health-related purposes, which is comparable to previous research that has found a large proportion of patients (70% to 90%) had access to email and interest in using email to communicate with their health care provider [14,16,17,20]. Increasing interest and openness to electronic communication highlights the “technological revolution” that has occurred in everyday life for patients [1]. The disinterest in text messaging and concerns regarding privacy in our survey respondents has likely lessened as a result of increased utility of mobile devices in general publication since the time of this study, as well as the improved public perception and comfort with health-related use of information technology [24]. Our study indicated that despite concern for confidentiality, 74.2% (194/334) of these patients would still allow for email communication. Those respondents who checked their personal email more frequently were also more likely to want to engage in health-related email communication. These individuals may have technology and electronic communication more fully integrated into their daily lives, such as through the use of mobile devices. However, those patients who did not have a personal email and who were not interested in engaging with their health care providers electronically represent an important demographic, who must not be left behind in this “technological revolution.” Interestingly, of the respondents who check their email less frequently than every 3 days, 45.7% (37/81) would still be interested in communicating with the McMaster Family Practice via email. This finding may indicate that patients would be interested in making use of their email for specific purposes, such as for health-related communication and decisions.

### Implications for Practice, Policy, and Research

This study indicates that email communication could provide an important avenue for health-related information between interested patients and providers. When used to its highest potential, electronic communication could enhance convenience, access, information sharing, satisfaction, and quality of care. However, at its basic level, email communication can have an impact on allowing for electronic scheduling and appointment reminders, as well as the opportunity for clarification after a face-to-face encounter with a primary health care provider or a specialist. While this is an ideal outcome of this technology, it is crucial that the “technological divide” does not hinder patient experience [2,14,16]. For example, patients who do not have interest or access to regular email must be able to maintain relationships with their providers. While patients may become increasingly accepting of the use of technology in their health care encounters, regulations must be in place to ensure that confidentiality and privacy in email communication remains a priority.

### Limitations

There are four key limitations to this study that have been identified. Firstly, participants in the survey were derived from a convenient sample of consecutive patients who were at least 16 years of age and who were attending the family practice on the date of data collection. While the final sample size was just over 600 patients, future studies should randomly select and survey members of the general and patient population. Secondly, those patients who did not use email at all (eg, patients who may be older, in poor health or of lower socioeconomic status) may have been less inclined to participate and complete the survey, or may not have been part of the population able to attend the clinic. As such, selection bias may have occurred in data collection and may have influenced the findings of this study. Thirdly, the patient characteristics of this family practice, which is an academic practice in an urban setting, should be considered when generalizing the results of this study. This study provides pertinent information for email communication at the McMaster Family Practice and the generalizability of these findings to other contexts or populations should be carefully assessed. Finally, opportunities for improvement of the study questionnaire itself have been identified, including the use of a scaled response grade for the questions asking about willingness to receive email and text communication from the clinic (as opposed to the dichotomous “yes” and “no” used), and asking about access to mobile devices and Internet.

### Conclusions

Our survey found that the majority of participating patients have a functioning email address and are willing to use email for health-related communication with the McMaster Family Practice. The willingness to receive email communication was not significantly correlated with age, indicating that older patients were still interested in this health communication approach. Surprisingly, privacy was not a significant concern for many patients, despite privacy being a common potential issue discussed in previous literature. A wealth of research has demonstrated that effective communication between patients and providers may positively influence patient’s behaviors and well-being, including satisfaction with care, medication adherence, recall and comprehension of medical information, and even functional and physiological status [25-27]. Email communication between patients and their health care providers can serve as a viable resource to enhance dialogue both inside and outside of the clinic room. While research has shown that clinicians find email communication with patients useful for administrative purposes (eg, appointment bookings, invitations, and reminders for preventive care), future research should examine whether specific approaches (eg, integration into personal health record) would make email communication more desirable for patients and providers. Future research should also assess the influence of email communication on specific aspects of the patient-provider relationship (eg, patient literacy, shared decision-making) and the best practices to maximize the effectiveness and quality of email communication between patients and their health care providers.

## Appendix

Multimedia Appendix 1 Survey instrument administered to primary health care patients.
