# Supplementary material for: Email Between Patient and Provider: Assessing the Attitudes and Perspectives of 624 Primary Health Care Patients
Source: JMIR Med Inform. 2016 Dec 22;4(4):e42. doi: 10.2196/medinform.5853 (PMC5216256; doi:10.2196/medinform.5853)
Supplement: Multimedia Appendix 1 [file medinform_v4i4e42_app1.pdf]

McMASTER FAMILY PRACTICE COMMUNICATION SURVEY

Help us improve the quality of your care!

We are conducting a survey to assess the quality and efficiency of our current methods in contacting our patients, which is primarily by telephone and mail. The usefulness of e-mail communication is being assessed, for a variety of roles ranging from appointment reminders to providing information about illnesses that are relevant to you!

If you are older than 16 years of age, then you are eligible to fill out this survey. Please complete the survery in its entireity to your best ability. Clearly circle a single selection for each question below.

How satisfied are you with our overall means of contacting you (e.g. mail, telephone, etc.)?

Very Dissatisfied	Dissatisfied	Neither Dissatisfied or Satisfied	Satisfied	Very Satisfied

Do you have a personal email address?

Yes	No

How frequently do you check your email address?

At Least Daily	Every 2-3 Days	Every 4-7 Days	Once a Week	Less Than Once a Week

Would you be willing to allow McMaster Family Practice to contact you via email address?

Yes	No

How often have you forgotten to book or come for an appointment?

Never (0)	Rarely (1-3)	Occasionally (4-6)	Often (>=7)

Would you be interested in accepting text messages in place of emails?

Yes	No

Which of the following would you find useful communicated by email (check all that apply):

- ☐ Appointments/Follow-up Reminders
- ☐ Flu-shot Reminders
- ☐ Medical Information relevant to you

How beneficial do you think that email communication from the clinic would be to your care?

No Benefit at All	Minimal Benefit	Not Sure	Some Benefit	Very Beneficial

How concerned are you about the privacy of email?

Not Concerned	Somewhat Concerned	Not Sure	Concerned	Very Concerned

Are you concerned about receiving junkmail/spam?

Not Concerned	Somewhat Concerned	Not Sure	Concerned	Very Concerned

What are the first three digits of your postal code?

__ __ __

Are you currently:

Student	Employed	Unemployed	Retired

What is the highest level of education that you have completed?

Less Than High School	High School	Some College	Undergraduate	Postgraduate

What is your age?

____

What is your gender?

Male	Female

We welcome your comments

____

____

____
